# Food Insecurity and Dental Caries in Rural Mexican Populations

**DOI:** 10.1155/2023/6694259

**Published:** 2023-10-06

**Authors:** César Iván Romo-Sáenz, Erika Marlene Chavez-Reyes, Ricardo Gomez-Flores, María Norma González-Flores, Ricardo Sosa-Martínez, Norma Cruz-Fierro, Jose Elizondo–Elizondo, Myriam Angelica de la Garza-Ramos

**Affiliations:** ^1^Laboratorio de Inmunología y Virología, Facultad de Ciencias Biológicas, Universidad Autónoma de Nuevo León, San Nicolás de los Garza 66455, Mexico; ^2^Facultad de Odontología, Universidad Autónoma de Nuevo León, Calle Eduardo Aguirre Pequeño y Silao S/N, Colonia Mitras Centro, Monterrey 64460, Mexico

## Abstract

**Objective:**

Food insecurity (FI) is a priority for government and health organizations. Over 95% of the world's population has a carious lesion or will develop one before death. This study evaluated the association between FI and oral health in two rural communities in Chiapas, Mexico.

**Methods:**

The study was conducted with patients attending an oral health campaign for dental checkups. Data were collected between April and August 2017 using the Latin-American and Caribbean Scale of Food Security (ELCSA) and the International Caries Detection and Assessment System (ICDAS). We included 209 participants from Siltepec and Huehuetan, Mexico; 67% were women.

**Results:**

The results of the ELCSA were mild FI in 43% (*n* = 91), moderate FI in 22% (*n* = 45), and severe FI (*n* = 6) in 3%; 32% had food security. The ICDAS results were initial decay with a mean of 6.22, moderate decay with a mean of 1.81, and extensive decay with a mean of 1.77.

**Conclusions:**

FI is associated with dental caries, and food-insecure individuals have a higher probability of severe dental caries. In this study, the FI level was lower than in other rural populations in Mexico. Identifying these individuals and addressing the factors related to FI can be useful in the rural communities.

## 1. Introduction

Food insecurity (FI) is the lack of regular access to sufficient and adequate food for the household [1]. It is a priority for government and health organizations because of its social implications and impact on health [2]. Several scientific reports have found a correlation between FI and chronic illnesses [3–5].

Dental caries is the most prevalent oral cavity disease and an important public health concern. Over 95% of the world's population have a carious lesion or will develop one before death, making it one of the most frequent chronic diseases of modern man worldwide [6]. The interaction between etiopathogenic factors in dental caries and health determinants is complex. Therefore, economic, demographic, and social determinants need to be analyzed. Oral diseases are more frequent in low-income countries, particularly those with high-poverty rates [6, 7]. However, a decrease in dental caries in Latin-American and Caribbean children has been found, a change caused by changes in social and public health measures, the effective use of fluoride sources, and improved living conditions [8]. Fluoride is added to several products in Mexico, such as toothpaste, rinses, gels, and water and there is a national program known as the fluoridated iodized salt project [9].

In Mexico, 28.2% of households had moderate or severe FI. Moderate to severe FI was more frequent in rural (35.4%), indigenous (42.4%), and low-socioeconomic households (45.9%) [2]. FI also affects elderly adults (27.9%), and high-FI levels are present in individuals with depression and impaired activities of daily living [10]. Moreover, the percentage of elderly adults is estimated to increase from 6.2% in 2010 to 22.6% in 2050 [10, 11].

Identifying risk factors for caries is important because they are closely related to nutrition. These factors include food preparation and the consistency and texture of food, in addition to habits and cultural practices [11]. Therefore, it is paramount to understand the relationship between social determinants and caries, especially FI, because of its relationship with poverty and demographic factors. This study evaluated the association between FI and dental caries in two rural communities in Chiapas, Mexico that have an elementary diet since they usually consume foods they produce.

## 2. Materials and Methods

This descriptive, cross-sectional study of a convenience sample of 209 patients from 85 family groups was conducted in the communities of Siltepec and Huehuetan, Chiapas, Mexico. Participants were recruited during an ambulatory oral health campaign.

Data were collected between April and August of 2017 during the 2-week campaigns. The protocol was previously evaluated and approved by the Institutional Bioethics Committee with registration number SPSI-010613 and was granted clearance number 00132.

The inclusion criteria were minors with primary dentition, accompanied by family members, and adults with natural teeth. People who refused to answer the questionnaire were excluded. All participants older than 18 years signed informed consent, and underage participants assented after a responsible adult signed informed consent. We first assessed FI, after which an oral clinical examination was performed by the dental professionals, who were trained to assess caries with the International Caries Detection and Assessment System (ICDAS) method. Community assessments were performed during the campaign on different days.

**Figure 1:**
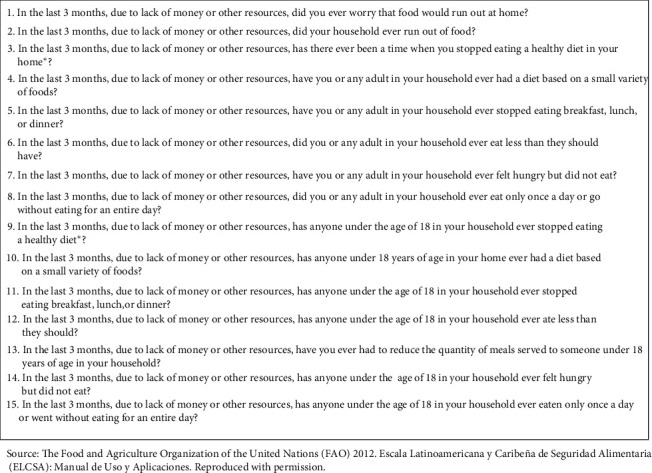
Latin-American and Caribbean Food Security Scale (ELCSA).

We assessed FI using the Latin-American and Caribbean Scale of Food Security (ELCSA). This scale was selected over other methods because of its sensitivity and specificity, being validated for this region of Mexico [12, 13]. The harmonized Mexican version of the ELCSA was applied because it provides information from the experience of family members. The ELCSA consists of 15 questions (Figure 1). The first eight refer to diverse situations that cause FI in adults in the household, and the remaining seven to conditions that affect members younger than 18 years. According to the protocol, only one questionnaire was applied per family.

The criteria for household FI evaluation were different for households with minors and households with members older than 18 years [13, 14]. The scale for households with minors was household food secure (Score 0), mild household FI (Score 1–5), moderate household FI (Score 6–10), and severe household FI (Score 11–15). For households with members over 18, the scale was household food secure (Score 0), mild household FI (Score 1–3), moderate household FI (Score 4–6), and severe household FI (Score 7–8) [15, 16] (Table 1).

The ICDAS was used to measure dental caries. ICDAS is a method that has been internationally adopted and applied [17]. Furthermore, this system provides better sensitivity for detecting carious lesions in different states of progress and activity than indexes, such as the DMF-T (Decayed, Missing, and Filled Teeth), which is also widely used [18]. The ICDAS is also used with caries management methods to control them. The system arranges carious lesions into the following groups: initial, moderate, and extensive decay [8, 16, 18].

Different operators in the different rooms performed questionnaires and oral examinations to avoid bias. The evaluators were trained to standardize the measurement process and ensure that the criteria in the evaluations were similar. After excluding children under 2 years of age, 209 people integrated into 85 family units participated. These families were diverse and included small children and elderly adults. Descriptive statistics were reported, and nonparametric correlations were calculated.

## 3. Results

**Figure 2:**
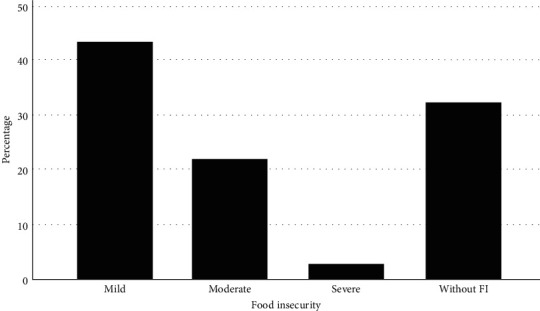
Percentage of food insecurity of 85 family groups according to the Latin-American and Caribbean Scale of Food Security (ELCSA).

The 209 participants from Siltepec and Huehuetan had an age range of 2–78 years, with a mean of 28.28. However, patients were stratified by age. More than 60% were women (67%), and the participants were integrated into 85 family groups, with a mean of 2.12 members per family and a maximum of five. ELCSA was calculated according to the presence of members younger than 18 years. Seventy families qualified with this criterion; the remaining 15 had only adult members. After calculation, the individual ELCSA measures were 43% mild household FI (*n* = 91), 22% moderate household FI (*n* = 45), 3% severe household FI (*n* = 6), and 32% household food security (Figure 2).

**Figure 3:**
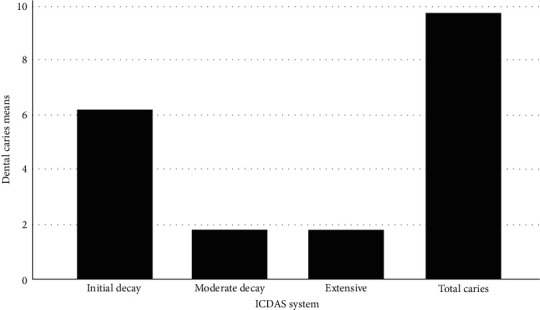
Dental caries means according to International Caries Detection and Assessment System (ICDAS) categories.

Dental caries was detected in 98% of the participants with the ICDAS system. The carious lesions varied from 0 (2%) to 27 (0.5%). The mean number of caries per patient was 9.81 (SD 5.48). Most lesions were classified as initial decay with a mean of 6.22. Moderate decay had a mean of 1.81, and extensive decay had a mean of 1.77 (Figure 3). The distribution of carious lesions varied according to the age group, with a prevalence of 85% in the 2–5 years age range. It decreased to 60% in those 6–12 years and increased to 100% in the rest of the age ranges (Table 2).

**Figure 4:**
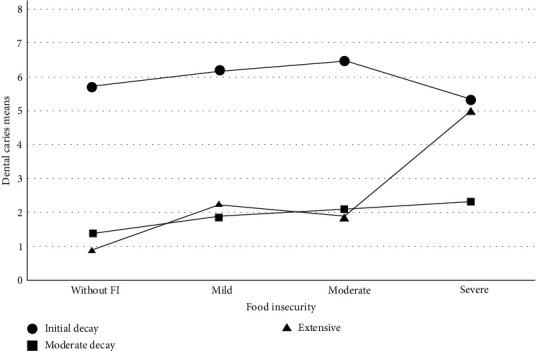
Relationship of dental caries means and food insecurity according to food security categories.

**Figure 5:**
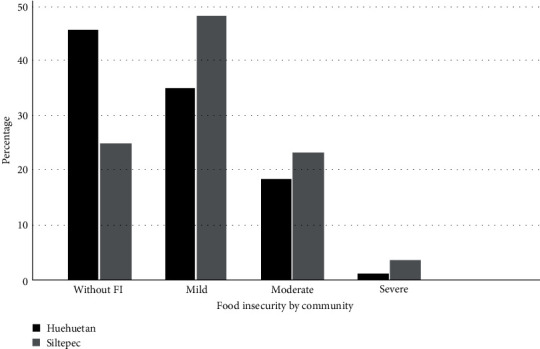
Comparison of percentages of food insecurity based on the ELCSA scale by community.

An association was found between household FI and the level of carious lesions. The distribution of initial and moderate decay remained stable in all FI categories. However, extensive decay increased in households with severe FI (Figure 4). We also found a correlation between FI and total caries in primary teeth, extensive cavities, and total caries. These results were statistically significant (*P* < 0.05) by the Rho Spearman test (Table 3). We also found a higher percentage of mild to severe FI in Siltepec (75%) compared to Huehuetan (55%) according to the ELCSA scale (Figure 5).

## 4. Discussion

FI was present in 67.14% of the individuals in this study. The most frequent FI was mild. This level of FI was lower than in other rural populations in Mexico. Many households in the communities reported some level of food production, either for commercial or personal use. However, this information was anecdotal, and further inquiry is needed to determine if this was a reason for the levels of FI. Other studies that have analyzed rural populations in Mexico reported FI ranging from 86.5% to 91% [1, 2, 5]. However, it is important to emphasize that in other studies, some questions were modified with regionalisms that might have affected the result.

Regarding basic information related to oral health teaching in school and basic dental knowledge education, 12 activities per child are carried out during the school year in preschool and 30 activities per child at the primary and secondary levels. These include bacterial plaque detection (4 during the school year), brushing technique instruction (4 during the school year), educational talks (4 during the school year), flossing instruction (4 during the school year), 0.2% sodium fluoride mouthwashes (14 during the school year), and educational talks (4 during the school year) [19].

Carious lesions were more prevalent in our study than in previous studies in the same population. A previous study of this population in 2015 reported a frequency of carious lesions of 95% [20]. However, the authors used a different system to assess caries that does not consider the initial carious lesion [17]. Reports of caries in rural populations in Mexico vary from 43% to 100% [7, 9–22]. Nevertheless, this variability may be due to using other caries assessment systems, with which most carious lesions are considered initial, dismissed, or underestimated. [9].

Nevertheless, the levels of carious lesions were expected for this group and were statistically significant when grouped by FI classification. In addition, the association between FI and extensive decay lesions might be related to the availability of highly processed food for households or the priority given to acquiring food over medical and preventive dental measures, such as consultations.

A limitation of this study was that it was conducted 6 years ago. The study had difficulties due to the geographical inaccessibility of the cities and the health restrictions implemented due to COVID-19. Another was the sample size because these communities are isolated and of difficult access, making it difficult to include a larger number of inhabitants. An assessment of an urban population would have been useful to compare results. Previous studies have compared FI to dental caries, particularly in children. In the United States, no association was found, whereas, in Brazil, an increase in untreated caries was reported without showing the impact on the severity of the carious lesions [4, 23, 24].

Although multiple procedures are available to restore decayed teeth [25, 26], there is a need for early diagnosis and social intervention to deal with FI.

## 5. Conclusions

The causes of food and nutrition insecurity are complicated. Most FI and nutrition problems are related to the social determinants of health, such as low income or unemployment, lack of access to nutritious food, affordable housing, and medical care. FI is associated with the dental caries. Addressing the factors that cause FI and providing population and individual interventions can prevent dental caries. Further research is needed to clarify the relationships and generalize this study's associations.

## Figures and Tables

**Table 1:** The criteria used to evaluate household food insecurity.

Criterion	ELCSA score <18 years	ELCSA score >18 years	Definition
Household food secure	0	0	Household has no problems or anxiety about access to food
Mild household food insecurity	1–5	1–3	Household is worried about not having enough food sometimes or are unable to eat preferred foods
Moderate household food insecurity	6–10	4–6	Household is forced to reduce the quality or quantity of the food they eat
Severe household food insecurity	11–15	7–8	The household has run out of food and gone a day or more without eating

**Table 2:** Distribution of caries by age range and International Caries Classification and Management System (ICCMS) category.

Age group (years)	Initial cavities	Moderate cavities	Extensive cavities	Total cavities	DMF-T	Prevalence (%) (DMF-T <0)
0–5	3.6	1.6	3.55	8.75	8.9	85
6–12	5.25	1.1	2.4	8.75	8.85	60
13–18	10.17	2.41	1.58	14.17	14.47	100
19–34	9.58	3	1.35	13.94	14.23	100
35–49	5.47	1.62	1.09	8.19	14.70	100
50–65	4.15	1.36	1.21	6.73	19.52	100
65+	4.28	2	0.85	7.14	20.57	100

*Note*: The total number of caries is the mean sum of all carious lesions. The Decayed, Missing, and Filled-Teeth index (DMF-T) is the mean, including missing and filled teeth. The prevalence of caries was calculated by counting a DMF-T higher than 0.

**Table 3:** Correlation of caries and food insecurity.

Nonparametric correlation with ELCSA	Spearman	Significance
Total number of cavities in primary dentition	0.178	^*∗*^0.01
Extensive cavities	0.269	^*∗*^0.000
Total number of cavities	0.200	^*∗*^0.04

*Note*: Differences were evaluated with the nonparametric Spearman correlation. The hypothesis of extensive decay is evenly distributed in the categories of FI and is rejected at <0.05 ^*∗*^.

## Data Availability

The data used to support the findings of this study are available from the corresponding author upon request.
